# La symphysite pubienne du post-partum: un diagnostic difficile

**DOI:** 10.11604/pamj.2013.16.14.3242

**Published:** 2013-09-13

**Authors:** Mehdi Kehila, Manel Majdoub, Dorra Zegha, Sonia Ben Khedher, Elhem Cheour, Sami Mahjoub

**Affiliations:** 1 Faculté de médecine de Tunis, Service C du Centre de maternité et de néonatologie de Tunis, Tunisie; 2 Faculté de médecine de Tunis, Service de Rhumatologie, hôpital la Rabta, Tunis, Tunisie

**Keywords:** symphysite pubienne, arthrite septique, accouchement, symphysite pubic, septic arthritis, delivery

## Abstract

La symphysite pubienne est une localisation rare de l'arthrite septique. Son association à la grossesse est exceptionnelle. Nous rapportons un cas de symphysite pubienne survenant dans les suites d'un accouchement par voie basse. Le diagnostic est difficile en post-partum. Il faut savoir l'évoquer devant toute douleur pubienne atypique dans sa présentation ou dans son évolution. Le diagnostic doit être posé de façon précoce car l'évolution peut se faire dans le cas contraire vers la chronicité.

## Introduction

L'arthrite de la symphyse pubienne est une greffe septique non spécifique de l'articulation du pubis. C'est une localisation infectieuse rare, survenant souvent chez des patients présentant un terrain particulier (chirurgie urologique et pelvienne, sportifs) [1]. Les symphysites pubiennes associées à la grossesse sont exceptionnelles [2]. Leur diagnostic est souvent difficile à cause de leur rareté et leur présentation souvent atypique pendant cette période. Nous rapportons ici un cas d'arthrite septique de la symphyse pubienne découvert en post-partum.

## Patient et observation

Il s'agit d'une patiente de 34 ans qui a été ré-hospitalisée à J15 du post-partum pour douleurs pubiennes et inguinales bilatérales avec impotence fonctionnelle des deux membres inférieurs. La patiente est 3^ème^ geste, 3^ème^ pare, sans antécédents pathologiques notables. Elle a deux enfants accouchés par voie basse de poids de naissance respectifs de 3600g et de 3650g. Le plus jeune âgé de 3 ans.

La dernière grossesse était de déroulement normal. La patiente a accouché à terme, par voie basse, d'un nouveau-né de poids de naissance 3750g. L'accouchement s'est déroulé sans incidents. Le lendemain de son accouchement, l'examen physique était strictement normal. La patiente a été mise sortante.

A J2 du post-partum, la patiente a décrit des douleurs inguinales bilatérales aigues, irradiant vers les fesses et la face interne des cuisses, s'aggravant à la station debout et occasionnant une boiterie à la marche, dans un contexte de fièvre non chiffrée. La patiente a consulté son médecin de famille. Le diagnostic de douleurs articulaires banales du post-partum a été d'abord évoqué et la patiente a été mise sous antalgiques et anti-inflammatoires. Toutefois, devant l'aggravation de l'impotence fonctionnelle devenant invalidante, la patiente nous a été réadressée à J15 du post-partum.

L'examen clinique à l'admission a trouvé une patiente apyrétique à 36,8 °C, une sensibilité à la palpation de la symphyse pubienne et à la mobilisation de la hanche surtout à l'adduction active. Le diagnostic d'une thrombose veineuse profonde du post-partum a été éliminé sur la base de l'examen physique, notamment devant la douleur exquise à la palpation de la symphyse pubienne. Un examen de la filière génitale sous valves a été pratiqué afin d'éliminer un hématome péri-vaginal. Il était normal. Le diagnostic d'une disjonction pubienne a été évoqué devant les éléments de l'examen physique malgré que l'absence de dystocie foeto-pelvienne lors de l'accouchement rendait ce diagnostic peu probable. La patiente a eu une radiographie standard du bassin qui a montré un élargissement de l'interligne articulaire de la symphyse pubienne de 10 mm et une irrégularité des berges cadrant avec le diagnostic de disjonction pubienne. Biologiquement il existait une hyperleucocytose modérée à 12 900 éléments/mm3, associée à un syndrome inflammatoire (CRP = 39mg/j).

**Figure 1:**
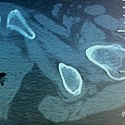
Diastasis de la symphyse pubienne associé à un aspect irrégulier des surfaces articulaires et cassure de la corticale

Après discussion du cas au staff, le diagnostic de disjonction pubienne paraissait peu vraisemblable devant l'absence de dystocie et de man'uvres lors de l'accouchement. Il a été alors décidé de compléter par un examen rhumatologique spécialisé. Celui-ci a suspecté une symphysite pubienne. Un examen scannographique a été demandé et a montré un aspect fortement évocateur de symphysite sans collection abcédée (Figure 1).

Le diagnostic d'arthrite septique du pubis a été de ce fait retenu. Un bilan infectieux étiologique a été réalisé et la patiente a été mise sous une antibiothérapie probabiliste à large spectre par voie parentérale associant une céphalosporine de 3ème génération et une fosfomycine, associée à des anti-inflammatoires non stéroidiens. Le prélèvement vaginal était positif à streptocoque B sensible à la Pénicilline. Les hémocultures et l'ECBU étaient négatifs. L'évolution a été marquée par une amélioration clinique progressive avec diminution des douleurs et de la boiterie au bout d'une semaine. La bithérapie a été maintenue pendant 8 semaines. Le syndrome inflammatoire biologique a disparu au bout d'un mois. La patiente était asymptomatique lors du dernier contrôle avec un recul de 6 mois.

## Discussion

L'arthrite septique de la symphyse pubienne est une affection rare représentant 0,8 à 1,36% de l'ensemble des arthrites infectieuses chez l'adulte [1]. Elle est exceptionnelle dans un contexte gravidique [3]. Il s'agit du premier cas rencontré dans notre service soit une incidence de 1/37580 accouchements.

Le diagnostic est classiquement évoqué devant une pubalgie fébrile compliquée d'une impotence fonctionnelle. La difficulté du diagnostic en post-partum réside, en plus de la rareté de la pathologie, dans le fait que cette période s'accompagne fréquemment d'algies et d'une symptomatologie articulaire variée, souvent difficile à analyser. En effet, l'inconfort des tables d'accouchement, le maintien prolongé en position gynécologique, l'élargissement physiologique de la symphyse pubienne lors de l'accouchement et l'épisiotomie sont souvent à l'origine d'une symptomatologie douloureuse ostéo-articulaire et périnéale en post-partum. En plus, la fièvre, le second symptôme utile au diagnostic, est souvent absente dans ce contexte [4]. Ce qui était le cas pour notre patiente.

Sur le plan biologique, il existe classiquement un syndrome inflammatoire avec une hyperleucocytose le plus souvent modérée. Cet élément est aussi difficile à analyser, car dans un contexte gravidique, une hyperleucocytose modérée peut être physiologique.

Tout ceci explique le délai, souvent long, entre le début des symptômes et le diagnostic (2 à 4 semaines) [2–4]. Chez notre patiente le diagnostic a été posé 13 jours après le début des symptômes.

Toutefois, malgré cette difficulté, après analyse critique de notre cas vécu et de quelques faits cliniques rapportés dans la littérature, on arrive quand même à isoler certains éléments cliniques qui doivent faire évoquer ce diagnostic. En effet, l'apparition secondaire d'une douleur qui n'existait pas en post-partum précoce, son intensité croissante, ou son évolution défavorable et non classique, la présence d'une fièvre ou d'une impotence fonctionnelle associée surtout après un accouchement non dystocique sont des éléments qui peuvent orienter le diagnostic et doivent par conséquent conduire à la réalisation d'un scanner ou d'une IRM du bassin. Ces examens montrent typiquement des érosions osseuses, un abcès des berges, un élargissement ou un épanchement de la symphyse pubienne [5]. Dans notre cas, devant un tableau clinique et biologique déroutant, un avis spécialisé et un scanner du bassin ont permis de poser le diagnostic.

La recherche du germe repose sur la ponction-aspiration, le drainage chirurgical d′une collection, ou le prélèvement au niveau d'une porte d'entrée. Dans la série de Ross, les hémocultures étaient positives dans 32% des cas et seulement 19% des patientes ont nécessité une ponction pubienne pour identification bactériologique [3]. Les staphylocoques dorés (34%) et les pseudomonas aeruginosa (24%) sont les germes le plus souvent incriminés [3]. Les symphysites pubiennes à streptocoque B sont exceptionnelles [3]. Dans le cas de notre patiente, les hémocultures étaient négatives. Le streptocoque B retrouvé sur le prélèvement vaginal rendait très probable son implication dans cette arthrite.

Le traitement repose, classiquement, sur une antibiothérapie d′au moins 6 semaines parfois associée au drainage d′une collection [3]. L′évolution est généralement favorable en cas de diagnostic et de traitement précoces, néanmoins 55% des patientes ont nécessité une intervention chirurgicale soit pour un débridement, soit pour l'évacuation d'un abcès [4]. En l'absence de traitement, l'évolution peut se faire vers la chronicité avec possibilité d'apparition de fistules, séquestres osseux et la possibilité d'une cellulite pelvienne [6].

## Conclusion

La symphysite pubienne est une pathologie exceptionnellement rencontrée dans le post-partum. Son diagnostic est difficile dans cette période. Il faut savoir l'évoquer devant toute douleur inhabituelle de la symphyse pubienne d'autant plus qu'elle est associée à une impotence fonctionnelle ou une fièvre. Le scanner et l'IRM sont d'une grande aide pour le diagnostic. Le traitement repose sur l'antibiothérapie prolongée par voie parentérale.
